# Headache and immunological/autoimmune disorders: a comprehensive review of available epidemiological evidence with insights on potential underlying mechanisms

**DOI:** 10.1186/s12974-021-02229-5

**Published:** 2021-11-08

**Authors:** Leonardo Biscetti, Gioacchino De Vanna, Elena Cresta, Ilenia Corbelli, Lorenzo Gaetani, Letizia Cupini, Paolo Calabresi, Paola Sarchielli

**Affiliations:** 1 Istituto Nazionale di Riposo e Cura dell’Anziano a carattere scientifico, IRCSS- INRCA, Ancona, Italy; 2 Section of Neurology, Department of Medicine and Surgery, University of Perugia, Perugia, Italy; 3 Headache Center, UOC Neurologia-Stroke Unit, Emergency Department, Ospedale S. Eugenio, Rome, Italy; 4 Department of Neuroscience, Università Cattolica Sacro Cuore, Rome, Italy

**Keywords:** Headache, Migraine, Autoimmune diseases, Immunological disorders, Neuroinflammation, Immune system, Cytokines

## Abstract

Several lines of evidence support a role of the immune system in headache pathogenesis, with particular regard to migraine. Firstly, alterations in cytokine profile and in lymphocyte subsets have been reported in headache patients. Secondly, several genetic and environmental pathogenic factors seem to be frequently shared by headache and immunological/autoimmune diseases. Accordingly, immunological alterations in primary headaches, in particular in migraine, have been suggested to predispose some patients to the development of immunological and autoimmune diseases. On the other hand, pathogenic mechanisms underlying autoimmune disorders, in some cases, seem to favour the onset of headache. Therefore, an association between headache and immunological/autoimmune disorders has been thoroughly investigated in the last years. The knowledge of this possible association may have relevant implications in the clinical practice when deciding diagnostic and therapeutic approaches. The present review summarizes findings to date regarding the plausible relationship between headache and immunological/autoimmune disorders, starting from a description of immunological alteration of primary headaches, and moving onward to the evidence supporting a potential link between headache and each specific autoimmune/immunological disease.

## Highlights

- Immunological events may be involved in the pathophysiology of primary headaches
- In many autoimmune diseases headache prevalence is higher than in general population
- For many autoimmune diseases headache may be a risk factor or a clinical manifestation

## Introduction

Headache is one of the most common neurological disorders and one of the most frequent reasons for medical consultation, both in primary care and in specialist settings.

Headaches are divided into primary—without an underlying cause—and secondary, those caused by other pathological conditions including trauma, haemorrhagic or ischemic lesions, tumours and infectious or inflammatory diseases. Clinicians need to consider all of the systemic and neurological signs, as well as laboratory findings, which are associated with headache, so to exclude a secondary form. The differential diagnosis between primary and secondary headaches is crucial, since it significantly influences clinical management, particularly from a therapeutic point of view.

Among secondary forms, headache has been suggested to be a neurological manifestation of many immunological/autoimmune disorders, both those primarily involving the central nervous system (CNS), such as multiple sclerosis, and those systemic, such as systemic lupus erythematosus. Based on this, over the last decades, researchers have focused their attention on better understanding possible links between headache and immunological/autoimmune diseases.

Both at epidemiological and pathophysiological levels, headache and immunological/autoimmune disorders seem to share common features. Most evidence in this field concerns migraine; thus, in this review, we will discuss mainly findings from studies specifically investigating a possible link between migraine and autoimmune diseases.

Epidemiologically, most immunological/autoimmune diseases are more frequent in females [1, 2]; likewise, almost all primary headache forms are more commonly diagnosed in females too, except for cluster headache [3]. Furthermore, the onset of both headache and immunological/autoimmune disorders are generally at young ages, with few exceptions, such as primary biliary cirrhosis and Graves’ disease [4].

In terms of pathophysiology, in primary headaches, and mainly in the migraine context, the role of both neuroinflammation and immune system derangement has been increasingly acknowledged in the last decades, further supporting the view of a link of these conditions with immunological disorders. Nevertheless, the relevance of a common pathophysiological background of headache and immunological/autoimmune diseases is still a matter of debate. Specifically, it is not clear whether headache is a direct, specific manifestation of a disease or a comorbidity, whether it is reactive to disease or its direct organic consequence and, most importantly, if headache, and in particular migraine, can predispose persons to the subsequent development of an immunological/autoimmune disorder.

## Neuroinflammatory mechanisms involved in headache

Nowadays, the relevance of the contribution of neuroinflammation in the pathophysiology of several painful conditions, including migraine, is widely accepted [5, 6]. Neuroinflammation could also be involved in pathophysiological mechanisms of cluster headache but evidence in this regard are limited and dated, whereas its contribution to pathophysiological events underlying tension-type headache is unlikely. Neuroinflammatory mechanisms have also been described in the context of several disorders causing secondary headache, such as post-traumatic stress disorder, chronic stress and traumatic brain injury [7, 8].

Specifically, pre-clinical models of migraine demonstrated that, a key role in its pathophysiology is played by the trigemino-vascular system activation, which typically induces a local neurogenic inflammation involving dural and pial vessels. This causes (i) a plasma protein extravasation due to an increased meningeal vascular permeability, and (ii) the activation of immune cells, namely resident mast cells and perhaps macrophages, localized near the dural afferents. Activated mast cells in turn produce several mediators including serotonin, histamine, heparin, proteases and arachidonic acid products, pro-inflammatory cytokines and chemokines. All these substances are strongly involved in the peripheral sensitization of trigeminal endings [9]. Sensitized trigeminal c fibres release calcitonin gene-related peptide (CGRP) which interacts with its own receptors on the dural vessels. This interaction determines vessel dilation by activating adenylate cyclase which is responsible for the increase of intracellular cyclic adenosine monophosphate and the decrease in intracellular Ca2+ [10]. In rodent models, CGRP also interacts with its own receptors on mast cells contributing to the release of inflammatory substances and cytokines, but this mechanism cannot be automatically translated to humans because of the lack of evidence of the presence of all components of CGRP receptors on human mast cells. Other neuropeptides such as pituitary adenylate cyclase-activating peptide or vasoactive intestinal peptide could play a role in mast cell activation and degranulation [11, 12].

Peripheral sensitization of the primary afferent trigeminal ganglion (TG) neurons leads to the subsequent central sensitization of trigeminal nucleus caudalis (TNC) second-order neurons which in turn induces the sensitization of third-order neurons in the thalamus. The maintenance of sensitization of neurons in structures involved in the processing of trigemino-cervical nociception is believed to drive the progression from episodic to chronic migraine [13, 14].

In the TG, CGRP binds to A delta TG neurons expressing CGRP receptors facilitating nociceptive transmission to second-order neurons in the TNC. Resident glial cells and astrocytes also possess CGRP receptors. The interaction of CGRP with its receptors on these cells induces the release of some pro-inflammatory cytokines such as tumour necrosis factor (TNF)-α and Interleukin (IL)-1ß which dramatically amplifies trigeminal nociception [15, 16].

The upregulation of pro-inflammatory cytokines—specifically IL-1ß—in activated microglia has also been shown in the TNC. Using different models of trigeminal activation, the nuclear factor kappa-light-chain-enhancer of the activated B cell (NF-Κb) signalling pathway and activation of NLR family pyrin domain containing 3 (NLRP) inflammasome were found to play a role in neuron-glia cross-talk contributing to the central sensitization [17–20].

Neuroinflammatory events involving activated microglia and astrocytes also occur in the course of cortical spreading depression (CSD), which is considered the pathophysiological substrate of migraine with aura (MA). Several preclinical studies revealed that CSD not only induces glial activation but also increases the expression of pro-inflammatory cytokines, adhesion models and chemokines as well the expression of toll-like receptors (TLR3 and TLR4) [21–25]. A neuroimaging study on MA patients, using a radioligand binding the 18-kDa translocator protein, a marker of glial activation, showed increased uptake in areas involved in nociceptive processing such as the thalamus primary/secondary somatosensory cortex and insular cortex as well as in areas primarily involved in CSD generation such as the visual cortex. This increased uptake appeared to be related to the frequency of attacks [26].

## The role of the derangement of the immune system in primary headaches suggested pathophysiological links with immunological/autoimmune disorders

Immunological and autoimmune disorders include several diseases or conditions caused by a dysfunction of the immune system.

Specifically, autoimmune diseases are characterized by abnormal immune responses to self-antigens resulting in a damage or dysfunction of a wide range of body tissues. Autoimmune diseases can be systemic or can affect specific organs and/or body systems. Their aetiology is multifactorial, involving both genetic and environmental factors. Likewise, the aetiology of headaches and specifically of migraine is accepted as being multifactorial.

Polymorphisms of genes encoding for human leukocyte antigens (HLA) and cytokines are considered risk factors for autoimmune disorders. Some studies have found that the same genes are involved in migraine pathogenesis [27, 28]. These findings suggest that a common genetic background renders patients more susceptible to both migraine and immunological disorders and that some immunological alterations, for instance changes in inflammatory cytokines levels, may have a role in migraine pathophysiology.

In this regard, a significant increase in the peripheral levels of pro-inflammatory cytokines such TNF-α, IL-1β, IL-6 and IL-8—whose involvement in many autoimmune disorders is well known—have been found in migraine patients, both in interictal and ictal periods [29–32]. The increase of the above cytokines and chemokines in migraine, especially between attacks, suggests a pro-inflammatory status underlying migraine regardless of the acute phase of the disease, which could possibly explain an association between migraine and some inflammatory/autoimmune diseases.

The increase of IL-10 levels observed during an attack in some studies [33–35] has been interpreted as a compensatory mechanism aimed to antagonize pro-inflammatory cytokines during the ictal period, by exerting anti-nociceptive effects and limiting neurogenic inflammation. This anti-inflammatory cytokine plays a pivotal role also in immune regulation and therefore may prevent immunological/autoimmune disease onset and progression [36, 37].

Like in several autoimmune diseases, an impairment in natural killer (NK) cells, as well as a significant increase in the CD4+ lymphocyte and a decrease in the CD8+ lymphocyte subsets, was observed in migraine patients [38–41]. Changes in CD4+ and CD8+ lymphocyte subsets were associated with a reduction of the immunoregulatory CD4+CD25+ cell levels in migraineurs in a recent study, suggesting that the failure of self-recognition mechanisms might play a role in migraine pathogenesis and predispose migraineurs to immunological/autoimmune disorders [42].

Theoretically, the prevalence of pro-inflammatory cytokines over anti-inflammatory mediators, as well as dysregulation of lymphocyte subsets in migraine, could furnish another pathophysiological explanation of the potential link between this primary headache and immunological disorders.

Even for cluster headache (CH), some evidence supports a role for immunological dysfunctions in the pathogenesis of this disorder. Dated findings in cluster headache include a negative association with HLA-B14, the increase in NK cytotoxicity, the augmented receptor expression of classical neurotransmitters of pain—such serotonin or histamine—on immunocompetent cells, and the increase in the levels of cytokines with a potent pro-inflammatory activity such as IL-1β [43]. Based on these observations, a robust association between CH and immunological disorders would have been expected. However, a study including 27 CH patients and 99 healthy controls failed to detect a higher prevalence of systemic inflammatory diseases in the CH group compared to controls, examining both laboratory and clinical data [44].

Little is known in the role of a derangement of the immune system in tension-type headache (TTH). Research in this regard revealed an increase in peripheral levels of IL-8 [45], IL- 6 [46] and IL-1 ß [47] and an increase in IL-1ra and monocyte chemoattractant protein-1 levels in the cerebrospinal fluid (CSF) [48]. Starting from these observations, one could speculate that pro-inflammatory cytokines might be associated with pain by their direct binding to receptors on afferent neurons, including peripheral myofascial nociceptors that are thought to be crucial in TTH. The activation of nociceptors by cytokines, in turn, appears to be able to generate action potentials, thus inducing pain hypersensitivity [49].

In conclusion, the plausible role of immunological mechanisms in the pathogenesis of primary headaches, and particularly of migraine, has been consequently suggested to represent a potential link between headache and some immunological/autoimmune disorders. Below, we will summarize the available evidence on the possible association between headache and each specific immunological/autoimmune disease, considering both epidemiological and pathophysiological aspects.

## Headache and multiple sclerosis

Multiple sclerosis (MS) is the most frequent immune-mediated inflammatory disease of the CNS, causing myelin loss and axonal pathology at variable degrees, therein generally leading to progressive neurological dysfunction and disability [50, 51]. Headache and migraine are common features in MS. Their occurrence can influence MS treatment and significantly impair the quality of life of patients due to related disabilities [52, 53].

### Epidemiological evidence

The first association between MS and headache was described in 1960 [54, 55]. Subsequently, several studies assessed the prevalence of migraine and TTH in MS patients and reported rates between 2 and 67% for migraine [56–74] and between 12.2 and 55% for tension-type headache (TTH) [61, 67]. This latter primary headache seems to occur more frequently in progressive form of MS. [61] Particularly for migraine, the occurrence was considerably higher in females. Furthermore, available evidence supports a bidirectional relationship between migraine and MS, highlighting the role of migraine as potential risk factor for MS. [75, 76]

The most relevant results of studies on the prevalence of headache in MS patients are shown in Table 1. The variability of the results in this regard can be in part attributed to the different criteria used for both MS and headache. Interestingly, in some of these studies, headache appeared to be correlated to MS subtypes [61, 67].

**Table 1:** Main relevant studies on the prevalence of primary headaches in MS patients

Authors and year of publication	Study design	MS pts Number (***N***) ***F***: % Mean/median age or age range (years)	Case source	Controls Number (***N***) F: % Mean/median age (years)	Control source	MS diagnosis criteria	Headache diagnosis criteria	Pts Migraine prevalence (%)	Controls Migraine prevalence (%)	Pts Other headaches	Controls Other headaches
Watkins & Espir 1969 [55]	Case-control	*N* = 100 (*F*: 64%) Age range = 15–50 years	Hospital	*N* = 100 (*F*: 64%) Mean age: 15–20 years	Hospital	Mc Alpine (1961)	Critchley definition for migraine (1967)	27%	12%		
Zorzon et al. 2003 [75]	Case-control	*N* = 140 (*F*: 64%) Mean age: 42.1 years	MS centres (mostly)	*N* = 131 Age and sex matched	Blood transfusion centre	Mc Donald et al. (2001)	NA	13.6%	0.7%		
Vacca et al. 2007 [66]	Case-Control	*N* = 238 (*F*: 65%) Median age: 40 years	Hospital (mostly)	*N* = 238 (*F*: 65%) Median age: 43 years	Friends	Mc Donald et al. (2001)	ICHD 2004	MwA: 31% MA: 3.7% PMwA: 6.3%	Mwa: 13% MA: 2.5% PMwa:2.9%	ETH: 6.3% CTTH: 0.8	ETTH:5.9% CTTH:0.0%
Nicoletti et al. 2008 [64]	Case-control	*N* = 101 (*F*: 64.4% Mean age: 43.6 years	Cohort	*N* = 101 (*F*: 60.3%) Mean age: 35.4 years	General population selection through random digit dialling	Poser (1983)	ICHD 1988	19.8%	15.8%	TTH 27.7%	TTH: 16.8%
Putzki et al. 2009 [65]	Case-control	*N* = 491 (*F*: 68%) Mean age: 45.3 years	Hospital (mostly)	*N* = 447 (*F*: 69.1%) Mean age: 45.4 years	Historical controls from German Headache Study	Poser (1983) or Mc Donald et al. (2001)	ICHD 2004	24.6%	39.9%	TTH 37.2%	TTH 34.4%
Kister et al. 2010 [62]	Cross-sectional case control	204 (*F*% NA) Mean age: 45 years	MS care centre	*N* = 162,576 (*F*% NA) Mean age: NA	AMP population	Mc Donald et al. (2001)	ICHD 2004	46.1%	11.7%	TTH: 28%	NA
Kister 2012 [76]	Prospective cohort study (period: 1989–2005)	*N* = 542 (*F*: 100%) 140 prevalent cases 402 incident cases Mean age: 45	NHS-II cohort (UK)	*N* = 97898 (non-MS people) (*F*% NA) Mean age: NA	NHS-II cohort (UK)	Mc Donald et al. (2001)	ICHD 2004	Prevalent cases 26% Incident cases: 29%	21%		
Simpson 2014 [73]	Case control	*N* = 3826 (*F*: 72.3%) Mean age: NA	Scottish Primary Care dataset	*N* = 1, 268, 859 (F: 51.1%) Mean age: NA	Scottish Primary Care dataset	NA*	Migraine = ≥ 4 only anti-migraine prescriptions in last year	OR 2.38; 95% CI 1.91–2.97)			
Gustavsen 2016 [71]	Cross-sectional case control	*N* = 510 (*F*: 73.6%) Mean age: 50.7 years	Oslo MS Registry	N = 914 (*F*: 58.3%) Mean age: 43.9 years	Norwegian Bone Marrow Donor Registry	McDonald revised (2011)	ICHD 2004	18.2%	16.3%	TTH 12.7%	TTH 14.9%

Studies included in this table were selected based on sample size (> 50 patients) and the presence of a control group

*MS was defined as the presence ever of a Read Code for MS using a code-set created by NHS Scotland Information Services Division (Scotland, I.S.D.N.N.S: Measuring long-term conditions in Scotland, June 2008. 2008. 02/02/14]

Abbreviations: *AMPP* American Migraine Prevalence and Prevention study, *ICHD* International classification of Headache Disorders, *MA* migraine with aura, *MwA* migraine without aura, *NHS* Nurses’ Health Study II, *pts* patients, *NA* not available, *TTH* tension-type headache

Based on previous published researches, a meta-analysis by Pakpoor et al. including 8 studies, for a total of 1,864 MS patients and 261,563 control cases, reported a significant association between migraine -including both MA and migraine without aura (MwA) subtypes- and MS (OR = 2.60, 95% CI 1.12–6.04), although with a significant heterogeneity [77]. However, when only MwA was considered, sensitivity analysis evidenced a significant association between this subtype of migraine and MS with an OR = 2.29 without a significant heterogeneity. A more recent meta-analysis including 11 articles and 12 abstracts conference papers found a pooled prevalence of migraine of 31.1% in a total of 11,372 MS cases, with a large variability among residents of different countries in the world [78].

Only one study specifically investigated a possible relationship between migraine and MS disease activity and observed that the migraine status in MS patients was significantly associated with a more symptomatic course of the disease, but not with a higher disability score or T2 lesion burden on brain magnetic resonance imaging (MRI) [62].

Additionally, other studies explored the occurrence of migraine or headache before MS diagnosis. One of these revealed the presence of headaches in 2/3 of patients before MS onset, half of them with a family history of headaches unrelated to familiar MS occurrence. Remarkably, aura was diagnosed in 64% of the patients with no prior history of headache until MS onset, and in 34% of the patients with a prior history of headache. In addition, patients who reported auras were more likely to complain of a worsening of headache during MS exacerbation [52].

Likewise, a prospective, multicentre investigation involving 50 patients with the diagnosis of clinically isolated syndrome (CIS) or MS, screened using a specific headache questionnaire, reported headache within the last 4 weeks in 78% of cases, with migrainous features in 50%. Interestingly, in the context of the first manifestation of MS, headache, especially with migrainous features, was more frequent than other symptoms or signs such as paresis, paraesthesia and hyperesthesia, optic neuritis, and brainstem or cerebellar signs. In this study, however, the lack of an appropriate control group did not allow for a definite conclusion on this high prevalence rate [79].

Indeed, based on published radiologically isolated syndrome (RIS) cohort studies, headache is by far the most common reason for performing MRI [80].

According to some authors, when MRI is performed specifically for headache and it evidences white matter (WM) lesions suggestive for MS, headache should be considered the first clinical manifestation of this inflammatory-demyelinating syndrome and accordingly screened. In particular, Gebhardt et al. suggested that patients with only headache and typical MS lesions could be classified as CIS or early MS, instead of RIS, and therefore could be treated with an immunomodulatory therapy, especially whenever new lesions are evidenced at neuro-radiological follow-up, even in the absence of clinical manifestations [79].

### Pathophysiological mechanisms

Some hypotheses can be advocated to explain the occurrence of headache and particularly migraine in MS.

**Fig. 1:**
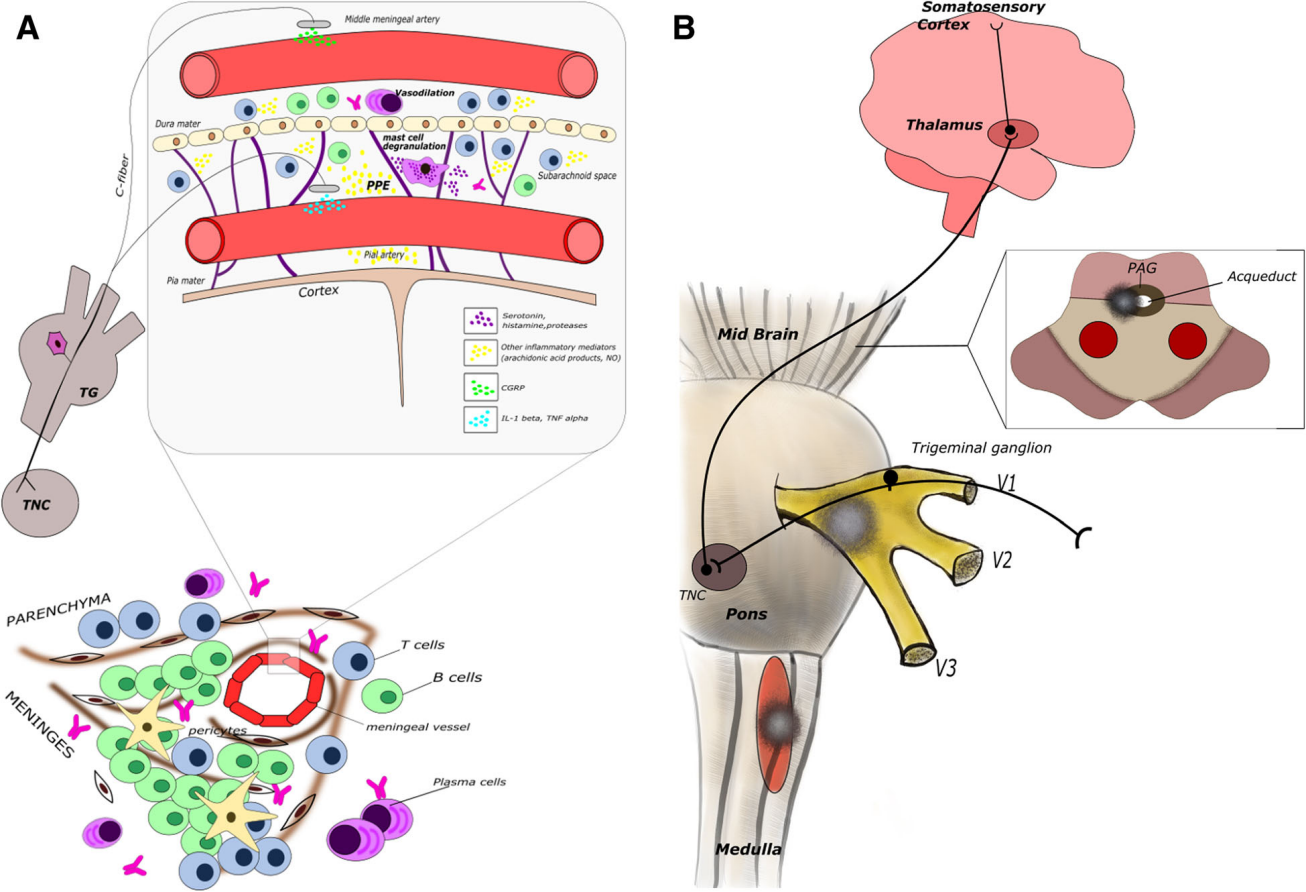
Putative CNS sites responsible for migraine or cluster headache-like pain in MS patients. Different sites could be involved in head pain generation in MS. **A** Lymphoid follicle-like structures in meninges can promote activation of trigeminal nociceptors (top of figures). This mechanism could be relevant for pain in both relapsing-remitting and progressive form. In the bottom of the figure, the mechanisms involved in trigeminal ending activation are shown. In particular, lymphocytes can produce proinflammatory cytokines, chemokine and other soluble mediators in the proximity of the meningeal and pial vessel wall which can promote sensitization of trigeminal nociceptors. The role played by CGRP released from trigeminal endings on T and B cell function in this context remain to be established. Mast cells can also be recruited and activated at the meningeal site. Mast cells possess multiple receptors for cytokines and chemokines and once activated secrete a large spectrum of preformed (early) and de novo synthesized (later) mediators including multiple cytokines and chemokines. Preformed mediators can mediate local vascular changes (PPE) and can promote further recruitment of other immune cells such as monocyte/macrophages and perhaps lymphocytes. Sensitized trigeminal endings convey nociceptive information from TG to TNC in the pons and from this nucleus to thalamic nuclei projecting to the primary somatosensory cortex, insular cortex, limbic structures and hypothalamus (not shown in the figure). Their activation is responsible for conscious perception of head pain as well as pain-related behaviours and autonomic responses. **B** Demyelinating lesions strategically located in different CNS sites related to trigeminal processing can be responsible for pain. Strategical sites include (i) the entry root site of trigeminal nerve in the pons, where the presence of a demyelinating lesion could be responsible for cluster headache-like or trigeminal neuralgiform pain; (ii) the spinal trigeminal nucleus where the presence of a demyelinating lesion may induce migraine or tension type-like headache; and (iii) the periaqueductal grey matter where the presence of a demyelinating lesion could cause a de novo migraine-like pain

Trigemino-vascular activation at the meningeal vessels is characterized by neuroinflammation at several sites of the trigeminal pathway, including meningeal vasculature, TG, TNC and subcortical cortical regions involved in head pain processing. Meningeal inflammation in MS, by causing the release of several pro-inflammatory cytokines, chemokines and end-products, such as nitric oxide, might provide a pathophysiological explanation for the high migraine prevalence in MS and its exacerbation during relapse [81]. This has been supported by evidence from post-mortem histological analyses of MS patient brains of both lymphoid follicle-like structures in the cerebral meninges and diffused meningeal inflammation related to the degrees of both microglial activation and grey matter cortical demyelination [82] (see Fig. 1). However, this finding was obtained from tissue blocks and whole coronal macro sections from a wide array of brain areas of secondary progressive MS patients, who, in epidemiological studies, more often refer headache with tension-type features, rather than with migraine-like ones. However, it cannot be excluded that meningeal inflammation was more relevant in MS patients in RR form particularly during relapse, but evidence in this regard is lacking at this time [81]. It cannot be excluded also that circulating pro-inflammatory cytokines or pro-inflammatory cytokines in CSF could contribute to the induction of headache with migraine-like features, but again, no studies are currently available in this regard.

Other mechanisms are deemed to be involved in the association between headache and specifically migraine and MS pathogenesis, such as a decrease in the CSF serotonin levels, a sympathetic hypofunction, and vitamin D deficiency [83, 84].

A further explanation for the occurrence of migraine de novo or the exacerbation of a pre-existing migraine is the location of lesions in strategic sites within the pathways involved in the processing of head pain. The relevance of demyelinating lesion location for the co-occurrence of MS and migraine have been supported by Gee et al. who reported that patients with at least a demyelinating lesion within the midbrain/periaqueductal grey (PAG) matter areas had a fourfold increase in migraine-like headaches, compared to MS patients without a lesion in these areas. This finding could be explained by the role attributed to PAG in the pathophysiology of migraine [85]. Conversely, the C2 location of demyelinating lesions has been associated with headache with tension-type features [65].

In the context of trigeminal neuralgia (TN), an underlying condition can be represented by an inflammatory lesion in the pons, even in patients without a definite diagnosis of MS. [86, 87] Furthermore, a recent study reported that, in patients with TN secondary to MS (TN-MS), differently from patients with TN without MS, neurovascular contact does not play a significant role in the aetiology of pain. Indeed, in TN-MS the primary cause seems to be demyelination along the intra-pontine trigeminal afferents. Therefore, the authors concluded that microvascular decompression should generally not be offered to TN-MS patients [88].

Likewise, a demyelinating lesion in the pons at the trigeminal nerve root entry zone might be responsible also for typical CH attacks, instead of a trigeminal neuralgic pain which more frequently occurs [89].

### Effect of disease-modifying treatment on headache/migraine

Disease-modifying treatment for MS can also influence migraine course in MS patients affected. Some studies in this context evidenced that interferon-β (IFN-β) seems to exacerbate migraine in MS patients who had already been suffering from this primary headache or induce a de novo migraine in patients without a previous history of headache [90–92]. Headache referred by patients treated with IFN-β has often migraine-like features and some of the patients compelling headache then develop medication-overuse headache and seek help from a physician due to the severity and frequency of headache [92]. Headache has also been reported as a side effect of fingolimod, teriflunomide and alentuzumab treatment, but reports from clinical trials investigating efficacy and safety on these immunomodulatory/immunosuppressive agents did not clarify the clinical characteristics of headache referred by patients [68, 93, 94]. Conversely, a longitudinal analysis of MS patients cohort revealed a significant reduction in migraine frequency for the subgroup of patients who were switched from IFN-β to natalizumab, irrespective of their level of fatigue, anxiety, depression, alexithymia or other clinical variables as well as Migraine Disability Assessment questionnaire scores [95, 96]. Headache is also rarely referred during dimethyl fumarate treatment [97]. It is noteworthy in this regard the evidence that this medication, when delivered via intraperitoneal injection in an animal model of nitroglycerin-induced migraine, exerts a protective effect on central sensitization by nuclear factors, including erythroid2-related factor (NRF2 ) [98]. These latter findings suggest that, among first-line therapies, dimethyl fumarate might offer relief for patients with both migraine and MS.

### Management of headache in MS patients

Management of headache and specifically of migraine in MS patients should be based on a combination of pharmacological and non-pharmacological approaches, as in the non-MS population. Specifically, migraine attacks can effectively be treated with triptans or non-steroidal anti-inflammatory drugs. Preventive drugs should be carefully selected taking into account their known efficacy/side effect profiles as well as any patient comorbidities. Patients with MS and headache should be instructed on the risk of symptomatic drug overuse, stressful conditions which can trigger attacks, and how to achieve more regular sleeping patterns. The presence of depression and/or anxiety in MS patients with headache significantly impair quality of life and it should be incorporated into the overall patient management [99].

Anti-CGRP and anti-CGRP receptor antibodies are the first tailored treatment for migraine and have been approved for the preventive treatment of high-frequency episodic migraine and chronic migraine [100]. Furthermore, CGRP antagonists are under evaluation for their symptomatic and prophylactic use in migraine [101].

One of the debated issue at this time is the use of these novel therapeutic strategies for migraine in patients affected by autoimmune diseases, specifically MS. [102]

Whether experimental evidence supports the role of CGRP as a potent trigger of acute neuorinflammation, its chronic tonic release, on the other hand, seems to exert immunomodulatory and anti inflammatory effects, by inhibiting innate immune response and limiting tissue damage [103]. Furthermore, this neuropeptide is strongly involved in host surveillance [104].

Prolonged antagonism of CGRP might therefore lead to a pro-inflammatory state or facilitate viral, bacterial and parasitic infections. However, the impact of blockade of CGRP on the overall immune balance at the moment is difficult to forecast. Thus, CGRP-blocking therapeutics should be carefully considered when used in patients with autoimmune diseases, such as MS, and close patient monitoring is recommended.

### Final remarks

Taken together, the above epidemiological findings support an association between headache, and in particular migraine with MS, already at its onset or immediately before and during the course of disease, especially in the relapsing-remitting phase of the disease. Pathophysiological mechanisms above reported furnish a potential explanation of this association but some of them, such as the implication of meningeal inflammation, need to be confirmed in future research.

## Headache and vasculitides

Vasculitides include a wide group of complex immunological diseases characterized by a relevant inflammation of blood vessel walls. An association between headache and vasculitides has not been definitely established, with some relevant exceptions. Of these, headache is considered a core symptom of giant cell arteritis (GCA). Furthermore, some evidence supports a correlation between headache and primary angiitis of the central nervous system (PACNS) [105]. Systemic vasculitides can also cause secondary headache, and Behҫet’s syndrome seems to be one of the most strongly associated with headache [106]. From a pathophysiological point of view, vasculitides leading to headache can affect the blood vessels that either irrigate the brain and meninges, or drain blood from these structures; both favour the release of CGRP and pro-inflammatory cytokines responsible for headache.

### Giant cell arteritis

#### Epidemiological evidence

Headache in GCA occurs at least over 70–80% of the time of disease and is typically severe. It may be unilateral or bilateral and is usually located in the temporal areas. Patients often describe the pain as sharp or burning, constant and severe and refractory to analgesia [107, 108]. Headache belongs to the category of symptoms due to the involvement of cranial vessels, followed by jaw claudication (pain on chewing), scalp tenderness, loss of vision and abnormalities of the temporal artery (pain on palpation, nodules, absence of pulse) [107–109]. Being so, headache is considered the most common symptom referred by GCA patients and the American College of Rheumatology include it among the diagnostic criteria of GCA (1990).

#### Pathophysiological mechanisms

GCA is a vasculitis targeting medium- and large-size arteries and is characterized by an inflammatory cellular infiltrate mainly CD4+ T cells and macrophages with giant cells in the media and interruption of the elastic lamina of vessels involved. The typical lesions, granulomas in the vessel wall, are formed by CD4+ T cells, which undergo in situ activation in the adventitia, where they interact with indigenous dendritic cells. Tissue injury is mediated by several distinct sets of macrophages that are committed to diverse effector functions [110]. The first studies investigating cytokine expression by patient’s temporal artery biopsies demonstrated an increased expression of IL-1, IL-2, IL-6, TGF-1 and IFN-γ [111]. Cytokines, such as Il-1ß, TNF-α and IFN-γ are the main inducers of adhesion molecule expression and are released from endothelial cells and from activated lymphocytes and macrophages [112]. Finally, the altered distribution of B cells in GCA has been related to CGA pathogenesis via the enhancement of IL-6 response [113]. Taken together, these immunological alterations may induce neuroinflammation and subsequently headache.

### Primary angiitis of the central nervous system

#### Epidemiological evidence

PACNS is a rare form of vasculitis with an estimated annual incidence rate of 2.4 cases per 1 million person-years. In a retrospective study of 101 patients with PACNS, the median age at the diagnosis was 47 years and 50% of patients were between 37 and 59 years at diagnosis [114]. Headache in PACNS can be moderate or severe at onset, having a chronic course ab initio or significantly worse over time. The characteristics and location of headache, are non-specific, like in many other forms of CNS vasculitis. Neck pain can be also associated.

Whenever there is suspicion of PACNS, reversible cerebral vasoconstriction syndrome (RCVS) should be excluded. PACNS and RCSV are frequently grouped together, given their shared clinical and radiological features. Headache is present in upwards of 60% of patients with PACNS and almost 100% in RCVS [115, 116]. In PACNS, headache is the most common symptom, followed by altered cognition and persistent neurologic deficits [117–119], while RCVS typically presents with acute onset of thunderclap headache with or without other neurologic symptoms [120]. Differential diagnosis between ‘true’ vasculitides and RCVS is pivotal, because the current anti-inflammatory medications used for the treatment of vasculitides can worsen the course of RCVS [105].

#### Pathophysiological mechanisms

Typical histopathologic features in PACNS are multifocal eosinophilic vascular infiltrates and necrotizing arterial wall damage, without evidence of amyloid deposition [121]. A granulomatous pattern was seen in 50% of a review of surgical biopsies [122]. Vessel alterations and inflammatory products from infiltrating cells can induce activation of nociceptive trigeminal endings distributing to brain vessels and can be responsible for headache. Less common complications, including ischemic strokes consequent to vascular thrombosis and intracerebral haemorrhage, can also be responsible for a new-onset headache, sometimes with the characteristics of thunderclap headache [123].

### Behçet's syndrome

Behçet's syndrome is a systemic vasculitis where thrombosis or thrombophlebitis involves small and large veins while arterial involvement is less frequent.

#### Epidemiological evidence

With regard to Behҫet’s syndrome, a study conducted by Kidd [106] on an unselected group of patients reported a high prevalence of headache (85.2%) with most of them suffering from headaches meeting the International Classification of Headache Disorders (ICHD) criteria for migraine. Moreover, the prevalence of visual or sensory aura was higher than that recorded in the general population of migraine suffers. The reason for this high prevalence of headache in patients with Behҫet’s syndrome is not fully understood.

#### Pathophysiological mechanisms

From a pathophysiological point of view, Behҫet’s syndrome can be considered a systemic ‘vasculitis/perivasculitis’. In this context, pro-coagulant factors, such as tissue factor, fibrinogen, thrombin and protein C, are believed to cause systemic inflammation and thrombus formation. Reactive oxygen species (ROS)-derived modifications also contribute to vascular structural and functional changes [124].

In CNS involvement by Behçet's syndrome, a clear typical vasculitis is uncommon [125], while the most frequent abnormality is represented by a marked perivascular cuffing of mononuclear cells, in particular T lymphocytes and monocytes, surrounding the small vessels [126]. In this case, microthrombotic mechanisms as well as oxidative stress might perhaps play an underlying role in fostering headache onset via activation of trigeminal nociceptive fibres distributing to affected small vessels [106, 127]. More difficult is to try to explain headache occurrence in patients with Behçet's syndrome without any clinical and radiological signs of CNS involvement. In this regard, it can be hypothesized that an enhanced inflammatory response, and the systemic overexpression of pro-inflammatory cytokines, in particular TNF-α, can contribute to the sensitization of trigeminal nociceptors like in other systemic inflammatory diseases [128], but data in this field are still lacking.

### Other types of vasculitides

#### Epidemiological evidence

Other types of vasculitides, including polyarteritis nodosa (PAN) and Takayasu’s arteritis (TAKA), can also involve CNS, resulting in cerebral infarctions and both intracerebral and subarachnoid haemorrhages, all of which may be responsible for secondary headaches [105]. TAKA is a large vessel vasculitis which typically affects young females. Headache usually occurs in the chronic phase of disease while it is rare in children [129]. In a recent case series of 67 patients with TAKA, 42.9% had neurological involvement and dizziness was the most common neurological complaint (74%) followed by visual disturbances (59.3%) and headache (55.6%) [130].

PAN typically affects medium vessel walls and is characterized by the formation of arterial pseudoaneurysms formed by erosion of the arterial walls via a necrotizing process. Neurological involvement most commonly presents with peripheral neuropathy as multiplex mononeuropathy and, less frequently, with CNS involvement. Ten percent of patients with PAN are at risk of ischemic strokes and cerebral haemorrhages [131]. Headache occurs in about 35% of PAN patients. It usually is referred later in the disease course, but sometimes, it is associated with early stages with core symptoms. PAN can mimic GCA, with headache and/or jaw claudication [132–134]. In a study on 27 patients with systemic necrotizing vasculitis and temporal artery biopsy localized vasculitis, Genereau et al. found that 22 of them (81%) had cephalic symptoms including jaw claudication (33%). Four patients who had temporal artery biopsy involvement led to an initial misdiagnosis of GCA, but later developed systemic symptoms that revealed the correct diagnosis of PAN [135].

Finally, small vessel vasculitides, such as Churg-Strauss Syndrome, Wegener Granulomatosis and microscopic polyangitis, are generally accompanied by polyneuropathy. CNS involvement in these diseases is rare, and their association with headache has not been well established.

#### Pathophysiological mechanisms

TAKA is a large vessel vasculitis characterized by a relevant infiltration of pro-inflammatory T cells in the vessel wall [136]. Abnormal immunity may play a pivotal role in its pathogenesis [137–139]. A recent transcriptome analysis in TAKA patients demonstrated a dysregulation of several genes in CD4+ and CD8+ samples compared to controls. Among these dysregulated genes, the most relevant included JAK/STAT and cytokine/chemokine-related signalling which therefore might be a promising target for treatment [140]. Headache as well as dizziness in TAKA has been attributed to the involvement of the vertebral artery, which in turn activates head nociceptive input to the brain [141]. In addition, it has been hypothesized that hypertension due to renal artery involvement especially observed in young patients may play a role in headache occurrence [142, 143]

#### Final remarks

Headache is a common finding in vasculitides and probably recognizes pathophysiological mechanisms specific for each of them. These mechanisms, however, are still not clear and need to be further investigated in the next years.

## Headache and connectivitis

### Systemic lupus erythematosus

Systemic lupus erythematosus (SLE) is a chronic systemic autoimmune disease, affecting the joints and multiple organs including the skin, heart, lungs, kidneys and nervous system. Neurological involvement—central, peripheral and autonomic nervous system—and psychiatric events in SLE is a subcategory termed ‘neuropsychiatric lupus’ (NPSLE) [144].

#### Epidemiological evidence

Neuropsychiatric symptoms affect about half of the patients with SLE over the course of disease. Their spectrum can vary from mild to severe and therein can negatively influence the prognosis of the disease, as they are a relevant cause of morbility and mortality [145].

Neuropsychiatric symptoms can also be among the earliest manifestations of SLE; indeed some reports have suggested that up to 40% of these symptoms appear during the first year from SLE diagnosis [146]. Caucasian ethnicity and older age are reported to be associated with shorter time to neuropsychiatric damage [147, 148].

The most frequent SLE manifestations of CNS involvement include headache, mood disorders and cognitive dysfunction, followed by stroke and seizures [144]. Specifically, headache has been reported as the most frequent symptom of NPSLE. Headache in some cases may present features suggestive of an underlying organic cause and these can include high severity, concomitant with other neurological symptoms, abnormal laboratory results, as well as a better response to corticosteroids compared to other currently available treatments.

The role of headache in SLE has been recognized by the inclusion in the SLE Disease Activity Index (SLEDAI) of ‘lupus headache’ as a descriptor, defined as a severe, persistent headache which is often of migraine type and unresponsive to analgesia [149]. In light of this, when the third edition of the ICHD-3 is considered, it would appear that the ‘lupus headache’ could be allocated to various headache subtypes, depending on additional clinical features, including new daily persistent headache, high-frequency episodic migraine or chronic migraine with or without medication overuse.

Several epidemiological studies have been performed over the last two decades evidencing a broad range of variability in the prevalence of headache and specifically migraine in SLE [150–165]. In Table 2, findings from studies on the prevalence of headache in SLE patients are summarized.

**Table 2:** Main relevant studies on the prevalence of primary headaches in SLE patients

Authors and year of publication	Study design	SLE pts Number (***F***%) Mean age (years)	Case source	Controls Number (***F*** %) Mean age (years)	Control source	SLE diagnostic criteria	Headache diagnostic criteria	Pts Headache (***H***) prevalence Migraine (***M***) prevalence	Controls Headache (***H***) prevalence Migraine (***M***) prevalence	Pts Other headaches prevalence	Controls Other headaches prevalence
Markus 1992 [155]	Prospective study	*N* = 90 (*F*: 95%) Mean age: 42.3 years	Community in Nottingam District	N=90 (F 95%) Mean age: 42.6 years	Patients’ relatives and medical staff	NA	*Blau definition	*M*: 34%	*M*: 17%		
Sfikakis et al. 1998 [162]	Case-control	*N* = 71 (*F*: 91.5%) Mean age: 37 years	Hospital	*N* = 89 Age-and sex-matched	Representative sample of the Greek population	ACR	Questions**	*H*: 32% *M*: 8%	*H*: 30% *M*: NA	TTH: 24%	TTH: 1.4%
Fernandez Nebro et al. 1999 [160]	Case-control	*N* = 71 (*F*: 91.5%) Mean age 36.6 years	Hospital	*N* = 71 Age and sex-matched	Healthy subjects accompanying non-lupus patients or other outpatients	ACR	ICHD 1988	M: 22.5% MA: 4.2%, MwA18: .3%	M: 18.3% MA 1.4%, MwA: 16.9%	TTH: 23.9%	TTH: 23.9%
Whithelaw D. 2004 [164]	Case-control	N= 85 (*F*: 95%) Mean age: 28.7	Hospital	*N* = 62 (*F*: 93%) Mean age:23.1 yrs	Nurses	ACR criteria	ICHD 1988	*M* prior SLE: 11% *M* with onset of SLE 38%	*M* prior to nursing : 7% *M* with onset of nursing: 10%	**Prior SLE** Stress *H*: 15% Sinus *H*: 1% Other *H*s: 1% **With onset of SLE** Stress *H*: 11% Sinus *H*: 0% Other *H*s: 13%	**Prior to nursing** Stress *H*: 38% Sinus *H*: 0% Other *H*s: 8% **With onset of nursing** Stress *H*: 66% Sinus *H*: 6% Other *H*: 13%
Lessa et al., 2006 [165]	Case-control	N=138 ( F: 98.3%) Mean age: 36 years	Hospital	N= 92 (F: 83.7% Mean age: 46 years	Hospital Pts with diffuse connective tissue diseases other than SLE	ACR	ICHD 1988	*H*: 75.7% *M*: 66.1%	*H*: 66% *M*: 52.2%	TTH: 13.9%	TTH: 16.3%
Katsiari et al., 2011 [166]	Case controls	*N* = 72 (*F*: 96.5%) Mean age 38.3 years	Naval hospital	SLE *N* = 72 (*F*: 96.2%) Mean age 37.8 years MS *N* = 48 (*F*: 96.5%) Mean age 35.9 years	Hospital	ACR	ICHD 2004	**SLE** *M*: 21% **MS** *M*: 23%	*M*: 22%	**SLE pts** Frequent TTH: 15% Chronic TTH: 15% **MS pts** Frequent TTH: 19% Chronic TTH: 8%	Frequent TTH: 16% Chronic TTH: 1%

Studies included in this table were selected based on sample size (>50 patients) and the presence of a control group

*Blau Jn (1984) Towards a definition of migraine headache Lancet I: 444-445

**All patients and controls were first screened on the basis of the question: ‘Have you suffered from severe headache during the last year?’, and if the answer was positive for at least one episode of headache every 2 weeks, she or he was considered a headache sufferer.

Abbreviations: *ACR* American College Rheumathology, *ICHD* International Classification of Headache Disorders, *M* migraine, *MA* migraine with aura, *MwA* migraine without aura, *MS* multiple sclerosis, *SLE* systemic lupus erythematosus, *NA* not available, *TTH* tension-type headache

Like in MS, discrepancies regarding prevalence results are most likely due to differences in study designs, study populations and methodological approaches, as well as the differing definitions of headache and headache subtypes adopted and, in some cases, the lack of control groups. Some authors described MA with a high frequency in patients with SLE [163], but other studies have not confirmed this finding [154, 160, 165].

Concerning TTH in patients with SLE, some investigations have revealed a higher prevalence of TTH compared to migraine [162, 167]; in additional studies, however, either an equal [154, 155, 158, 160, 168] or higher [156, 161, 163, 169] prevalence of migraine compared with TTH was observed.

In 2004, results of a meta-analysis by Mitsikostas et al. did not find any differences in headache prevalence between controls and SLE patients, when considering the total population of SLE patients, with and without neuropsychiatric manifestations [170]. In agreement with this, a 2011 study by Katsiari et al. observed a similar prevalence of migraine (MA and MwA), among SLE patients (21%), MS patients (23%), and controls (22%). However, in this study, the duration and severity of migraine attacks were milder in SLE patients compared to controls. Only chronic TTH was significantly more prevalent in SLE patients (12.5%) compared to controls (1.4%). Furthermore, either in SLE or MS patient groups, no associations between any headache type and clinical manifestations, autoantibodies or disease activity were found [166].

In 2011, Unterman et al. [146] published a meta-analysis that specifically investigated the prevalence of neuropsychiatric manifestations in SLE. The authors found a 12.2% prevalence of headache in the pooled cohort and 23.3% (28.3% in a random-effects model) when only prospective studies were considered. These results are therefore in apparent disagreement with those by Mitsikostas et al. who reported a 50.2% prevalence in the pooled group of SLE patients. This finding is worthy of some consideration. The low prevalence of headache in Unterman et al.’s meta-analysis can be explained by the inclusion of a large Asian population who are known to report a lower prevalence of headache. Another factor of inhomogeneity of the results is the inclusion in Unterman et al.’s meta-analysis of both studies with a retrospective and prospective design with lower prevalence of headache in retrospective chart-driven studies; patients in these latter studies account for 3008/5057 patients in the entire cohort. Indeed, when analysing the prevalence of headache only in prospective studies excluding one on Chinese subjects and one where headache was not recorded, Unterman et al. calculated a 37% estimated prevalence using a random-effects model, a result which is much closer to the 50.2% prevalence found by Mitsikostas et al. Notably, a similar prevalence (52%) was found also by a recent meta-analysis involving paediatric SLE populations [171].

In line with the evidence of a higher estimated prevalence of headache in prospective studies than in retrospective ones (especially when the former imply longer follow-up), Hanly et al. in a prospective international inception cohort study, involving 1732 SLE patients, found at the baseline 17.8% of patients with headache (60.7%, migraine, 8.6%, tension-type headache, 7.1%, intractable non-specific headache, 2.6%: cluster headache and 1.0%. intracranial hypertension), but the prevalence of headache increased to 58% after 10 years. In this study, only 1.5% of patients specifically met the criteria of lupus headache, as identified in the SLEDAI [172].

#### Studies evaluating the association between headache and SLE activity

In the context of research investigating the association between headache and SLE activity, a study carried out by Markus and Hopkinson evidenced a close temporal relationship between the onset of both headache and SLE in many patients and that both migraine and non-migraine headaches often respond to specific SLE treatments [155]. In addition, Amit et al. observed that SLE patients experiencing moderate to severe headache on at least two consecutive encounters, additionally suffered from more severe joint pain, muscle pain, photosensitivity, mouth ulcers, fever and fatigue compared to SLE patients without headache. Patients with headache also had higher disease activity scores, and many of them showed CNS involvement [173].

Appenzeller and Costallat also revealed that SLE patients with active migraine had higher SLEDAI scores compared with those without. The authors went on to state that SLE patients with a history of migraine had also significantly higher cumulative organ damage scores compared to SLE patients without. Furthermore, active migraine was associated with higher levels of antiphospholipid antibodies and worsening of Raynaud’s phenomenon [174]. In contrast with the above results, other studies were not able to verify an association among disease activity (i.e. SLEDAI-2K scores with or without lupus headache), damage scores (i.e. Systemic Lupus International Collaborating Clinics (SLICC)/American College of Rheumatology Damage Index-SDI scores), use of corticosteroids or antimalarials or immunosuppressive medications, and either occurrence or frequency of headache with particular regard to migraine. In most cases, headache has been reported to be unrelated to other SLE manifestations and has its own independent course, which is generally recurrent or chronic [166, 170, 172].

Based on these conflicting findings, lupus headache was not included in the American College of Rheumatology Ad Hoc Committee definition of neuropsychiatric syndrome in SLE.

#### Pathophysiological mechanisms

The pathogenic mechanisms of NPSLE and ‘lupus headache’ remain to be elucidated. Numerous autoantibodies have been detected in plasma samples of SLE patients and have been linked to NPSLE. They include anti-ribosomal-P, anti-DNA/NR2, anti-DNA (16-1 idiotype), antiphospholipid (aPL), anticardiolipin (aCL) and GABA antibodies [175, 176]. It has been hypothesized that auto-antibodies or circulating pro-inflammatory cytokines/chemokines cross the blood–brain barrier (BBB), therein entering the brain and inducing neurotoxicity [177–180].

As part of research investigating autoantibodies profile, Hawro et al. reported that in NPSLE patients, the presence of autoantibodies to β2GPI were significantly associated with non-specific intractable headaches, ischemic stroke and seizures, and resulted having a better predictive value than either aCL or lupus anticoagulant (LA) [181].

Mechanisms possibly implicated in the pathogenesis of neuropsychiatric manifestations of SLE are complex. They involve genetic factors, vascular damage and occlusion, a BBB dysfunction, neuronal damage mediated by autoantibodies or inflammatory mediators including cytokines, and also a direct neuronal cell death [182]. Two relevant pathogenetic pathways have been identified: (i) a prevalent ischemic-vascular mechanism induced by aPL, immune complexes and leuko-agglutination involving large and small blood vessels, which is considered more frequently responsible for focal neuropsychiatric manifestations, and (ii) a predominantly inflammatory-neurotoxic mechanism mediated by complement activation, increased permeability of the BBB, intrathecal autoantibodies migration as well as local production of immune complexes and pro-inflammatory cytokines and other inflammatory mediators, which possibly account for neuropsychiatric diffuse manifestations.

However, if this latter mechanism could explain headache in some SLE patients remains to be established.

Findings on the specific clinical features and laboratory abnormalities associated with headache and migraine in the course of SLE are also inconsistent. CSF levels of different cytokines have been reported to be lower in SLE patients with migraine compared to SLE patients with other neuropsychiatric manifestations [183] and similar CSF IL-6 concentrations compared to controls. Even the levels of autoantibodies known to be involved in SLE were not clearly associated with headache [150, 156].

Neuroimaging studies based on MRI [184, 185], H1-Magnetic resonance spectroscopy [186] and single-photon emission computed tomography [187, 188] have been also performed in order to identify the anatomic and functional correlates underlying the possible association between SLE and headache. Unfortunately, these studies were not able to definitely clarify this issue due to the small sample size and in some cases the lack of a control group [188]. Furthermore, results of the above morphometric MRI, MRS and SPECT studies have not been replicated and therefore abnormalities detected could not be currently assumed as a specific flag of the association of headache and SLE.

#### Final remarks

A link between SLE and headache remains therefore controversial. Indeed, on one hand, headache is considered by many authors the most frequent neuropsychiatric manifestation of SLE, but on the other hand, the prevalence of headache in the total SLE population is overall similar to that observed in the general population. Furthermore, neither laboratory findings nor results of conventional and non-convention neuroimaging studies furnish specific and confirmed hallmarks supporting a clear association between these two conditions.

In addition, neuropsychological factors could also influence the occurrence of headache in SLE patients. These latter tended to present a high prevalence of anxiety and depression, suggesting that headache in SLE patients may, at least in part, be induced by emotional stress related to the clinical status [162, 175].

Therefore, based on the available evidence, in patients with SLE headache does not itself require further investigation, but should be classified according to IHS criteria and, in most cases, be managed as a primary headache.

### Primary Sjögren’s syndrome

Primary Sjögren’s syndrome (pSS) is a chronic, autoimmune disease characterized by dryness of both the mouth and the eyes associated with the involvements of other exocrine glands, along with a multiplicity of organs and systems [189]. Mononuclear cell infiltrate and progressive injury of the exocrinee glands are the main pathological hallmarks of the disease. The direct etiopathogenetic roles of the antiRo (SSA and SSB) antibodies have been defined. The prevalence of the disease ranges between 1% and 3% in the general population (female/male ratio about 9:1 in the age group 40–50 years) [190, 191]. When associated with other autoimmune rheumatic diseases, it is defined as secondary Sjögren’s syndrome (sSS), representing 30% of cases.

#### Epidemiological evidence

Several epidemiological studies, as well as pathophysiological and histopathological research, have emphasized the involvement of the Peripheral Nervous System in Sjogren’s syndrome (SS), whereas the CNS involvement has not been fully defined. Since the first observation in 1982 by Alexander et al. of focal or diffuse symptoms suggesting CNS involvement, later studies have been performed to investigate the possible occurrence of neuropsychiatric syndromes also in seronegative forms of SS [192–194].

Since the adoption of the current diagnostic criteria for SS in 2002 [195], little agreement has been reached regarding the prevalence of CNS signs and symptoms, ranging from 2.5 to 60% [196–199]. This variability may be influenced by the lack of a universally shared definition of CNS involvement in primary Sjögren’s syndrome (pSS). Moreover, neurological onset may sometimes precede both the clinical appearance of systemic symptoms and the immunological diagnosis by many years [200]. Thus, a pSS should always be considered in patients with relatively non-specific neurological symptoms, such as headaches, associated with sicca syndrome [201]. Among CNS manifestations of pSS, headache seems to be one of the most common. According to a study by Morreale et al. [196], the most frequent type of headache observed in a cohort of pSS patients fulfilled ICHD-II criteria for MwoA, while TTH subtypes and chronic or medication overuse complications were less frequent.

Other epidemiological studies have not well established a relationship between headache and SS. While some of them reported a significantly higher prevalence of migraine in patients with pSS compared to controls [202, 203], in contrast a population-based retrospective cohort study did not evidence any difference in the prevalence of migraine between patients and controls. In this latter study, chronic TTH was the only form of headache which resulted more commonly in pSS patients when compared to healthy subjects. However, chronic TTH was not associated with pSS-related autoantibodies, fatigue, depression, abnormalities on MRI or abnormalities in the cerebrospinal fluid. Higher depression and fatigue scores emerged in patients with pSS, but they were not associated with headache [204].

Some researchers have also investigated the presence of dry eye in migraine patients. In particular, Koktekir et al. found significantly lower tear function test scores for migraine patients, compared to controls. However, the authors did not assess the correlation between dry eye severity and migraine pain scores [205]. Celikbilek and Adam revealed that the presence of dry eye was higher in migraine patients compared to normal controls, but the difference did not reach statistical significance. They also reported a higher prevalence of MA and longer duration of attacks in migraine patients with dry eye [206]. More recently, Sarac et al. observed a higher prevalence of dry eye in 50 migraine patients compared to age- and sex-matched healthy subjects. The authors speculated that dryness of the ocular surface may activate the trigeminal nerve to cause reflex lacrimation, and the activation of the trigeminal nerve may trigger migraine headache [207].

#### Pathophysiological mechanisms

Mononuclear cell infiltration leading to a progressive injury of the exocrine glands is the main pathophysiological feature of pSS. Immuno-mediated mechanisms (especially an anti-Ro/SSA-mediated small vessel vasculitis) can also involve the CNS and can explain some clinical manifestations of the disease [208]. In particular, headache, cognitive dysfunction, mood disorders and fatigue referred by some patients suggest a higher diffused CNS compromission rather than focal involvement such as in MS-like clinical course or optic neuromyelitis-like syndrome [209].

In this regard, Escudero et al. proposed that migraine-mimic headache in pSS could be a direct expression of the disorder and not a mere comorbidity, as suggested in the neuropsychiatric SLE [210].

In the study of Morreale et al [196], .MwoA occurrence resulted significantly related to SSA antibodies, MRS alterations (reduction of NAA levels or decrease in NAA/Cr ratio) and hemodynamic dysfunction at ultrasonographic evaluation, but not to the presence of vasculitic brain lesions and/or macrovascular damage (such as WM lesions or MS-like lesions). In addition, the frequency of headache and alterations at MRS was higher in patients with Raynaud’s phenomenon. According to the authors, these findings suggest a possible endothelial dysfunction of the cerebral microcirculation or a potential inflammation-mediated shift of the neurovascular coupling which possibly accounts for both headache (especially migraine) and Raynaud phenomenon. In other words, headache in pSS has been suggested to be related to an ‘autoimmune endotheliitis’ which directly alters biochemical and humoral markers, in turn inducing perivascular inflammation that fosters vasomotor dysfunction.

#### Final remarks

Headache is a frequent finding in pSS and probably recognizes a vascular inflammation and endothelium dysfunction at the basis of its induction.

### Scleroderma

Nervous system involvement in scleroderma has been increasingly recognized and an association between migraine and systemic scleroderma (SSc) has been suggested.

#### Epidemiological evidence

In a 1978 study by Goldberg et al., SSc with typical migraine headache occurred in 16 well-documented cases observed over a 25-year period. In 13 of these, SSc developed after 15 years or more of therapy with ergot or methysergide preparations [211]. Based on these findings, the authors suggested caution when administrating these drugs for patients with migraine and more so in presence of signs of Raynaud's disease or early vascular SSc. In a more recent study including 182 case reports of patients with SSc and 50 diagnosed with localized scleroderma (LS), CNS involvement was observed in a sizeable proportion of patients. The most frequent symptom was headache (23.73%), followed by seizures (13.56%) and cognitive impairment (8.47%). Depression and anxiety were also frequently observed (73.15% and 23.95%, respectively). In LS, seizures (41.58%) and headache (18.81%) were the most commonly reported symptoms [212].

Treatment of SSc with nervous system involvement is not yet standardized. However, corticosteroids and cyclophosphamide are usually prescribed in severe cases. The effect of these treatments on headache in SSc has not been investigated.

#### Pathophysiological mechanisms

No studies have been carried out investigating the mechanisms underlying the headache occurrence in scleroderma; therefore no conclusions can be drawn at the moment on this matter.

### Rheumatoid arthritis and other forms of chronic rheumatic disorders

Rheumatoid arthritis (RA) is a chronic disease producing inflammatory synovitis in multiple joints.

#### Epidemiological evidence

This immunological disease seems to be more prevalent in patients with migraine. In a questionnaire survey of migraine patients in Denmark, the prevalence of RA was significantly higher in migraineurs compared to patients without migraine [213]. Another population-based study of headache patients in the USA reported that migraineurs were significantly more likely to suffer RA (OR 1.95, C.I.95% 1.68-2.25) [214]. A recent study looked at whether patients who had migraine were more likely to later develop RA [215]. Nearly 58,000 patients with a diagnosis of migraine were compared with similar number of age- and sex-matched control subjects. During follow-up, 461 subjects in the migraine group in contrast with 220 in the non-migraine group developed RA. The incidence rate of RA was 3.18 per 1000 person-years in the migraine group and 1.54 per 1000 person-years in the non-migraine group. Furthermore, compared to the control group, the crude hazard ratio of RA for the migraine group was 2.15 and the multivariable-adjusted hazard ratio was 1.91.

Of late, a link between headache and rheumatic disorders has also been investigated in the paediatric population. Specifically, a cross-sectional study assessed the presence, prevalence and clinical characteristics of primary headaches in 601 paediatric patients with chronic rheumatic diseases, such as juvenile idiopathic arthritis (JIA) and familial Mediterranean fever (FMF), using a semi-structured 53 item headache questionnaire. A higher prevalence of migraine (29.1%) followed by TTH (13.8%) in patients with paediatric JIA and FMF was documented. Among primary headaches, migraine and probable migraine were observed in 67.0% of FMF and 69.4% of JIA patients, while TTH was observed in 32.9% of FMF and 30.6% of JIA patients. Overall, one-third of these migraine patients had a family history of migraine in parents [216].

#### Pathophysiological mechanisms

It has been hypothesized that the association between migraine and RA might be due to a shared pathogenic mechanism; namely a dysfunction of the serotonergic system [193]. Indeed, serotonergic dysfunction has been implicated in the pathogeneses of both RA and migraine. In this regard, Zeller et al. observed that platelet serotonin levels were significantly decreased in RA patients and at the same time were inversely related to clinical RA activity [217]. Furthermore, Sacre et al. reported that serotonin reuptake inhibitors reduced the production of inflammatory cytokines in human RA synovial membrane cultures [218]. Another study suggested that the susceptibility for RA is related to genetic polymorphisms of the serotonin receptor 2A [219]. Likewise, impaired serotonin metabolism was evidenced in migraineurs, possibly triggering cranial vasoconstriction and neuronal sensitization [220]. Serotonin depletion has also been associated with increased cortical neuron sensitivity and enhanced vascular responses induced by CSD in an animal model of migraine [221]. As far as JIA is concerned, a small vessel disease has been suggested to underlie the clinical manifestations of JIA and may contribute to microvascular dysfunction causing migraine.

### Antiphospholipid syndrome

Antiphospholipid syndrome (APS) is an autoimmune, pro-thrombotic, multisystem disorder characterized by the combination of several clinical manifestations including arterial and/or venous thrombosis, pregnancy complications and/or recurrent foetal loss, thrombocytopenia and the presence of persistently positive antiphospholipid antibodies (aPLs) [216, 222–224].

aPLs represent a diverse collection of autoantibodies, directed against membrane phospholipids, including lupus anticoagulant (LA), anti-b-2-glycoprotein I (b2GPI), prothrombin (PT) and cardiolipin antibodies (aCL). The principal targets for the aPLs are b2GPI and PT [225–227]. APS can be accompanied by additional clinical features, including valvular lesions, migraine, Raynaud’s phenomenon, livedo reticularis, arterial hypertension and autonomic disturbances, such as postural tachycardia syndrome, neurocardiogenic syncope and orthostatic hypotension. In clinical practice, APS is classified as primary (PAPS) or secondary (SAPS), according to the absence or presence of another autoimmune disease, respectively [228].

#### Epidemiological evidence

Among neurological complications, recurrent headaches are quite prevalent in APS patients. Migraine is the most common type of headache and the most frequent neurological manifestation of APS [229], ranging from classic intermittent MA or MwA to unremitting, debilitating headache [230]. Furthermore, a study by Schofield et al. reported that migraine was the most common clinical, not only neurological, manifestation (87%) whenever APS coexisted with different types of autonomic disorders [231].

Several studies investigated the prevalence of migraine in APS, detecting rates ranging between 0 and 30% [232–243]. Later data from the Euro-Phospholipid Project revealed a 20% prevalence rate of migraine in APS patients [229]. More in detail, all the studies conducted on the European population with APS documented the co-existence of headache with LA and/or CL [244, 245], except one involving a group of Serbian patients [246]. According to a study by Hughes et al. APS patients report the onset of headache in their teenage years, then often disappears for 1 or 2 decades, only to reappear in their 3rd to 4th decade [247]. Transient ischemic attacks (TIAs) and visual or speech disturbances are accompanying symptoms in some patients, and often there is a strong family history of headaches [245, 246]. Therefore, some authors have recommended screening for aPLs in patients known to have migraine or recurrent headaches, since there might be a link between migraine and stroke in APS patients [247–249].

A study carried out by Zhu et al. [250] on a cohort of 51 APS patients showed a higher prevalence of headache in those with neurological antiphospholipid syndrome (NAPS), when compared to the rheumatologic form of the disease (RAPS). Interestingly, the authors detected by digital subtraction angiography (DSA) in NAPS patients typically multifocal vascular stenoses. In particular, while gadolinium-enhanced MRI failed to detect any lesions, DSA clearly evidenced a bilateral lack of blood vessels in the parietal lobes of 9 patients.

Based on the observation of headache in patients with APS, further research focused on assessing the prevalence of APLs in migraineurs also in the absence of a clinical diagnosis of APS. Some studies on this matter reported a higher prevalence of aPLs in migraineurs, when compared to healthy controls [233, 237, 238], while others did not observe an association [236, 241, 251, 252]. This discrepancy has been challenged by a recent systematic review re-analysing 11 case-control studies published between 1991 and 2014. The authors found a significantly increased rate of APLs in migraineurs (*n* = 170/779, 21.8%), when compared to healthy controls (*n* = 63/741, 8.5%) (*p* < 0.0001) [253].

#### Pathophysiological mechanisms

As far as the mechanisms underlying headache in APS, it can be speculated that circulating APLs may damage endothelium of the vessels resulting in active thrombogenesis and slow fibrinolysis [254]. Endothelium and vascular wall alterations in the dural vasculature as well as inflammatory reactions and products from aggregated platelets may be suitable triggers for meningeal trigeminal ending activation, which is responsible for headache in some patients. However, the exact mechanisms underlying this association have not still been clarified.

#### Treatment considerations

Headache associated with APS is often untreatable, poorly responsive to analgesics and/or narcotics and sometimes starts several years before the diagnosis of APS. Therapeutic strategies such as oral direct thrombin or anti-factor Xa inhibitors, hydroxychloroquine, statins, B cell inhibition, complement inhibition, and peptide therapy have been proposed for the management of thrombotic APS. However, a simple approach to prophylaxis against CNS complications of PAPS and SAPS is anticoagulation [255, 256]. Indeed, heparin followed by long-term anticoagulation with warfarin remains the gold-standard treatment [257]. Specifically, anticoagulation can lead to a marked improvement or resolution of migraine in many cases [258, 259]. Moreover, corticosteroids alone or in combination with anticoagulants and other therapies, including immunosuppressive regimens, might be a beneficial treatment option for both PAPS and SAPS patients with headache.

#### Final remarks

Headache is a frequent clinical manifestation of APS. The best management of this condition probably includes anti-inflammatory and anticoagulants drugs, but randomized controlled trials will be necessary in this field in the next years to definitely clarify this issue.

## Migraine and immuno-mediated endocrine disorders

Studies investigating a possible relationship between immuno-mediated endocrine disorders and migraine are scarce and have prevailingly focused on type 1 diabetes mellitus (DM) and thyroid disorders.

### Diabetes mellitus type 1

#### Epidemiological evidence

Unlike other autoimmune diseases where a high prevalence of headache has been documented, a negative association between type 1 DM and headache has emerged from population-based cross-sectional studies. In the Head Hunt Study, the OR of migraine was lower among patients with DM, compared to those without, being 0.4 for type 1 DM and 0.7 for type 2 DM, respectively. In the same study, the prevalence of migraine among patients with diabetes varied depending on age, with a higher prevalence in younger age groups [260]. A more recent research specifically investigated for an association between type I DM and headache in particular migraine. From a multivariate analysis of 26,121 participants in 2 surveys, an OR of 0.55 emerged for headache and 0.47 for migraine, compared to those without DM after adjusting for age, gender, years of formal education, and smoking. Accordingly, the merged group of patients with type 1 DM and latent autoimmune diabetes of adults had a lower risk of migraine (OR of 0.53). Similar results were obtained from a third survey including 39,584 participants. In contrast with findings from patients with type 1 DM, this survey reported no inverse association between headache and type 2 DM [261].

#### Pathophysiological mechanisms

Probably, changes in vascular reactivity and diabetic neuropathy, as well as insulin treatment, might be involved in the lower prevalence of migraine in patients with type 1 DM [261]. Conversely, the lack of a clear inverse association between migraine and type 2 DM might suggest an involvement of different genetic and environmental factors implicated in type 1 and type 2 diabetes.

### Hypothyroidism

#### Epidemiological evidence

High frequencies of migraine in patients with either overt or subclinical hypothyroidism have been recorded. Accordingly, in a recent study, the lifetime prevalence of MA and MwA appeared to be significantly higher (46%) in subclinical hypothyroidism patients recruited from an endocrine clinic, when compared to age- and sex-matched controls (13%) with an OR = 5.80 (95% CI 3.35–10.34). No differences in the levels of thyroid hormones and antibodies emerged between patients with subclinical hypothyroidism with migraine and those without. Noteworthy, an increased risk of developing other autoimmune diseases was noted in patients presenting both subclinical hypothyroidism and migraine, when compared to patients with subclinical hypothyroidism without migraine [262]. Furthermore, young migraineurs had an increased risk of developing subclinical hypothyroidism; likewise, adult headache patients, in particular those with migraine, had an increased risk of developing new-onset hypothyroidism [263, 264].

#### Pathophysiological mechanisms

Like other autoimmune disorders, hypothyroidism is a complex disease deriving from an interaction between a specific genetic background and environmental factors, therein leading to the exposure of thyroid autoantigens, which can induce the development of thyroid peroxidase and/or thyroglobulin autoantibodies. However, the mechanisms underlying the occurrence of headache in patients with hypothyroidism are unknown and merits investigation.

## Headache and immuno-mediated gastrointestinal disorders

### Epidemiological evidence

Several studies reported a significant association between migraine and immuno-mediated gastrointestinal disorders especially celiac disease (CD) and inflammatory bowel disease (IBD) [265–267].

In a study involving 72 adult patients with biopsy-proven CD, screened for concomitant neurological disorders, 28% suffered from migraine often in association with other neurological symptoms [268]. Another recent research, involving 188 patients with proven CD, found that there was a significantly higher prevalence of migraine in this group compared to controls (OR = 3.79), particularly for females and for CD patients < 65 years old. A greater proportion of patients with CD or gluten sensitivity (GS) (62 and 60%, respectively) graded their migraine as severe, compared to patients with IBD (30%) [269]. The link between migraine and CD suggested the benefit of a gluten-free diet for reducing migraine frequency in patients with both disorders. Accordingly, a preliminary study involving 90 migraineurs and 236 controls observed a CD prevalence of 4.4% in the migraine group vs 0.4% in controls. Overall, of the 4 patients having both migraine and CD, for one, migraine disappeared at 6 months of a gluten-free diet, while the remaining three reported a significant reduction in intensity and frequency of headache [270]. A subsequent Italian study indicated that migraine-type headaches were more common in CD patients than in controls and partially improved with gluten-free die [271]. Results from other cohorts of CD patients demonstrated similar improvements in migraine-type headaches whenever dietary intervention was recommended [272, 273].

A recent systematic review and meta-analysis study, including 40 articles published between 1987 and 2017, reported a mean pooled prevalence of headache among patients with CD equal to 26% (95% CI 19.5-33.9%) in adult populations and 18.3% (95% CI 10.4–30.2%) in paediatric populations. The headaches most often had migraine-like characteristics. In children with idiopathic headache, the prevalence of CD was 2.4% (95% CI 1.5–3.7%), whereas data for adult populations on this were unavailable [274].

In patients suffering from both CD and migraine, it is sometimes possible to observe WM abnormalities and cerebral calcifications on MRI, as well as deranged regional cerebral blood flow on SPECT can be present in some cases [275–277].

Faced with a higher prevalence of migraine in CD patients compared to controls, current evidence does not clearly support the adoption of a routine search for IgA antitransglutamase antibodies within the diagnostic approach for patients with migraine [278, 279]. Only one cross-sectional study has reported a higher prevalence of positive tissue transglutaminase IgA (tTGA) antibodies in a group of paediatric migraine patients (5.5%), compared to age- and sex-matched controls (0.6%). However, in this study, most patients with positive antibodies had normal biopsies, thus challenging the veracity of CD diagnosis and therein possibly highlighting the low diagnostic accuracy of only antibody testing [280].

As for celiac disease, the prevalence of migraine in IBD patients has been shown to be significantly higher than in the general population (21.3% vs. 8.8%, *p* = 0.027), with attacks having a longer duration, but not a higher frequency, compared to controls [281]. Likewise, migraine prevalence resulted twofold higher in IBD patients compared to the general population in a subsequent investigation conducted by Moisset et al. In the same study, migraine was associated with younger age, female sex and higher depression scores. Although migraine impact was relevant for 30% of patients, specific acute treatments, however, were prescribed in only 22% of cases. The authors suggested that a systematic screening for migraine should be done by IBD specialists in daily practice to provide adequate treatment [282].

In a recent study involving 60,436 US adults aged ≥18 years participating in the 2015 and 2016 National Health Interview Survey (NHIS), a higher prevalence of age-adjusted migraine or severe headache prevalence was reported among participants with IBD than those without IBD (28.1% vs. 15.2%, p < 0.0001). This association [adjusted prevalence ratio (95% CI) = 1.59 (1.35-1.86)] awas maintained after controlling for all other covariates [283].

### Pathophysiological mechanisms

The pathophysiological mechanisms behind the association between migraine and immune-mediate gastrointestinal diseases (both CD and IBD) are not fully understood. One of the hypotheses which has been advanced to explain an association between CD and migraine sustains that there is a potential role of a generalized inflammatory response, rather than direct antibody-mediated mechanisms in the induction of migraine attacks via trigemino-vascular system activation [284]. Specifically, increased levels of pro-inflammatory cytokines such as IFN-γ and TFN-α might be involved in the modulation and release of CGRP from trigeminal endings, which are in turn implicated in neurogenic inflammation; thus, possibly explaining the appearance and progression of migraine in CD patients [285, 286]. A similar mechanism has been advocated to explain the association between migraine and IBD. A lack of vitamins and/or macronutrients might contribute also to an association between migraine and CD [267].

In both inflammatory mediated disorders—IBD and CD—a role for gastrointestinal microbiota has been suggested. Specifically, unbalanced gut flora might be associated with neurological diseases like migraine, that is dysbiosis and the increased intestinal permeability might contribute to enhanced pro-inflammatory response representing a potentially mediator of both disorders [267, 286, 287]. Furthermore, a dysfunction of the serotoninergic system might be implicated in the progression of IBD [288–290]. Since serotoninergic dysfunction is involved also in migraine [291, 292], it is possible that this neurotransmitter pathway might represent a link between these two disorders.

## Headache and allergic disorders

### Epidemiological evidence

Several studies have investigated for an association between migraine and atopic diseases, both in adult and paediatric populations. In particular, a coexistence between asthma and migraine-type headaches has been described as well as a greater prevalence of hay fever, rhinitis, and dermatitis in migraineurs compared to healthy non-atopic controls [293–299]. Furthermore, children had a higher risk of asthma whenever their parents have had a history of migraine, suggesting that the two disorders might derive from a common denominator [300].

Asthma has also been indicated as a risk factor for new-onset chronic migraine [301–303]. In most of these studies, however, diagnosis of allergic disorders was not definitive and was solely based on the medical histories and allergic or respiratory symptoms, without spirometric confirmation. The most recent findings on this topic regard the presence of migraine in atopic children and vice versa. Specifically, in a study by Wang et al., the incidence of migraine was 3.2-fold higher in a cohort of children with atopic rhinitis, compared to a matched cohort of children without. The risk was greater for males, and for those aged < 6 years, and highest within the first year after atopic rhinitis diagnosis [304]. A further study examining children with one or more previous allergic diseases (atopic dermatitis, allergic conjunctivitis, allergic rhinitis, and asthma) suggested that they had a greater subsequent risk of migraine compared to controls by the time they reached school age. Furthermore, with more allergic disease, a cumulative effect for the risk of migraine was observed [305]. In agreement with the mentioned results here, lower ‘degrees of atopy’ were associated with less frequent and disabling migraine headaches in younger patients, while higher degrees were associated with more frequent migraines.

Moreover, the immunotherapy administration seems to have induced a decreased prevalence, frequency, and disability of migraine headache in subjects with asthma < 45 years of age [306]. A recent case-control study conducted across three European tertiary care hospitals revealed that children and adolescents with migraine were more likely to have persistent asthma and that persistent childhood asthma was associated with a higher frequency of migraine attacks. Interestingly, a history of anti-asthmatic or anti-allergic therapies was associated with a decreased risk of migraine, suggesting a potential role of these medications for the prevention of migraine occurrence [307]. Given this, physicians should be more aware of the risks of migraine in children with allergic diseases. In such cases, it would be reasonable to screen young migraineurs to exclude the presence of any atopic disorders particularly asthma, especially in those with a positive family history of asthma.

### Pathophysiological mechanisms

Mechanisms underlying an association between migraine and atopic diseases remain a matter of debate. Common pathophysiologic pathways have been hypothesized. Specifically, platelet activating factor and vasoactive neuropeptides are thought to play a role in both asthma pathogenesis and the induction of migraine-type headache. Food sensitivities have also been suggested as possible triggers of migraine attacks. Indeed, not only respiratory allergens but also food allergens, such as red birch, hazel and olive trees, along with nettle and wheat, have been reported to lead to migraine attacks in some patients with positive allergy tests. In these patients, the frequency of migraine attacks was reported to be higher compared to negative ones [308]. Accordingly, skin prick tests and blood tests for IgE-specific food allergens have been suggested as useful tool for selecting patients with migraine who could likely benefit from an elimination diet [309]. However, in a cohort of 50 migraineurs tested by Pradalier et al., prick-tests and radioallergosorbent test resulted positive only for 4 and 6 patients (all of them atopic) respectively, thus suggesting a low possibility of an association between migraine and allergy to foods staples [310]. Although data are contrasting, IgG food sensitivity testing has also been proposed for patients with migraine who refer some foods as potential triggers for attacks [311]. This test might be useful in the view of providing tailored dietary recommendations for patients and in order to avoid, at least in some cases, the use of medications [310]. Moreover, an elimination diet based on IgG food sensitivity tests combined with probiotics has also been suggested to be beneficial for migraine patients with irritable bowel disease [312, 313].

## Conclusions

This comprehensive review summarizes the findings to date regarding the association between headache and immunological/autoimmune disorders. Although studies are quite conflicting, over the last three decades, evidence has been moving towards a possible confirmation of the comorbidity of headache with almost all systemic immunological or autoimmune disorders or brain-directed autoimmune diseases, with the exceptions being SLE and type 1 DM. In the former, there does not appear to be a strong association, while the latter appears to be even a protective factor for migraine. In Table 3, we summarize the strength of association between headache and immunological/autoimmune diseases.

**Table 3:** Association between headache and immunomediated/autoimmune diseases

Autoimmune disorders	Association with headaches
Multiple sclerosis (MS)	↑
Vasculitis	↑
Systemic lupus erythematosus (SLE)	=
Primary Sjögren’s syndrome (pSS)	↑/=*
Systemic scleroderma (SS)	↑**
Rheumathoid arthritis and other forms of arthritis	↑
Antiphospholipid syndrome (AP)	↑
Type 1 diabete mellitus (DM)	↓
Hypothyroidism	↑
Immunomediated gastrointestinal disorders	↑
Allergic disorders	↑

↑ association; = no association; ↓ inverse association

* Some positive results, other negative

** A few studies are available on the association with headache

As far as the mechanisms underlying these comorbidities is concerned, contrasting hypotheses have been advanced. For some immunological/autoimmune diseases, headache and particularly migraine, could be, at least in some cases, a direct consequence of the disease location at the level of CNS. For instance, in multiple sclerosis, demyelinating lesions in the brainstem might account for the occurrence of headache, especially of cluster-like headache or trigeminal neuralgia. Alternatively, the migraine phenotype might be a consequence of general inflammatory mechanisms involving meningeal vessels and activating trigeminal terminals, especially in individuals with a previous history of migraine. Furthermore, in patients with chronic autoimmune disorders, headache, especially with tension-type features, could be reactive to the psychological burden of the diagnosis. Finally, as with most headaches, several stressors, especially infections and emotional stress, can trigger autoimmune disease exacerbation.

Immunological/autoimmune disorders and headache tend to worsen each other. Indeed, on one hand, several immunological/autoimmune diseases during active phases can exacerbate headache; on the other hand, the presence of headache in patients with immunological/autoimmune disorders worsens their quality of life. Therefore, a tailored treatment plan based on the most appropriate choice of acute and prophylactic therapies is needed in patients with both headache and immunological/autoimmune diseases. In this view, stricter cooperation between headache specialists and rheumatologists needs to be achieved in order to improve patients’ quality of life.

## Data Availability

The authors searched data for this review article from the online archives Pubmed and Embase.

## Abbreviations

aCL: Anticardiolipin
aPL: Antiphospholipid
APS: Antiphospholipid syndrome
b2GPI: Anti-b-2-glycoprotein I
CGRP: Calcitonin gene-related peptide
CD: Celiac disease
CH: Cluster headache
CIS: Clinically isolated syndrome
CNS: Central nervous system
Cr: Creatine
CSD: Cortical spreading depression
CSF: Cerebrospinal fluid
DM1: Type 1 diabetes mellitus
DM2: Type 2 diabetes mellitus
DSA: Digital subtraction angiography
EDSS: Expanded Disability Status Scale
FMF: Familial Mediterranean fever
GCA: Giant cell arteritis
HLA: Human leukocyte antigens
IBD: Inflammatory bowel disease
ICHD: International Classification of Headache Disorders
IL: Interleukin
JIA: Juvenile idiopathic arthritis
LA: Lupus anticoagulant
LS: Localized scleroderma
MA: Migraine with aura
MCP: Monocyte chemoattractant protein
MRI: Magnetic resonance imaging
MRS: Magnetic resonance spectroscopy
MS: Multiple sclerosis
MwA: Migraine without aura
NAA: N-Acetylaspartate
NAPS: Neurological antiphospholipid syndrome
NK: Natural killer
NPSLE: Neuropsychiatric Systemic lupus erythematosus
PACNS: Primary angiitis of the central nervous system
PAG: Periaqueductal grey
PAN: Polyarteritis nodosa
PAPS: Primary antiphospholipid syndrome
PNS: Peripheral nervous system
pSS: Primary Sjögren’s syndrome
PT: Prothrombin
RA: Rheumatoid arthritis
RAPS: Rheumatologic antiphospholipid syndrome
RCVS: Reversible cerebral vasoconstriction syndrome
RIS: Radiologically isolated syndrome
RRMS: Relapsing/remitting MS
SAPS: Secondary antiphospholipid syndrome
SLE: Systemic lupus erythematosus
SLEDAI: SLE Disease Activity Index
SLICC: Systemic Lupus International Collaborating Clinics
SPECT: Single-photon emission computed tomography
SS: Sjögren’s syndrome
SSc: Systemic scleroderma
sSS: Secondary Sjögren’s syndrome
TAKA: Takayasu’s arteritis
TG: Trigeminal ganglion
TN: Trigeminal neuralgia
TNC: Trigeminal nucleus caudalis
TNF: Tumour necrosis factor
TN-MS: Trigeminal neuralgia secondary to multiple sclerosis
TPO: Thyroid peroxidase
tTGA: Tissue transglutaminase IgA
TTH: Tension-type headache
